# Tolerability of cariprazine in the early stage of schizophrenia: A pooled, post-hoc analysis of 4 phase ii/iii double-blind placebo-controlled trials

**DOI:** 10.1192/j.eurpsy.2021.1400

**Published:** 2021-08-13

**Authors:** Z.B. Dombi, K. Acsai, Á. Barabássy, B. Sebe, I. Laszlovszky, G. Vass, B. Szatmári, M. Patel, W. Earley, G. Németh

**Affiliations:** 1 Medical Division, Gedeon Richter Plc., Budapest, Hungary; 2 Global Medical Affairs, AbbVie, Madison, United States of America; 3 Clinical Development, AbbVie, Madison, United States of America

**Keywords:** Cariprazine, schizophrénia, antipsychotic, safety

## Abstract

**Introduction:**

In the early stage of schizophrenia (first 5 years), the most important clinical target besides symptom control is relapse prevention as each relapse significantly decreases the possibility of preferable long-term outcomes. Early discontinuation of antipsychotic medication due to intolerable side-effects is one of the most common causes of relapse.

**Objectives:**

This poster aims to present cariprazine’s tolerability in the early stage of schizophrenia.

**Methods:**

Data from 4 randomized, double-blind, placebo-controlled trials (NCT00404573, NCT01104766, NCT01104779, NCT00694707) with similar design (1 week of wash out period, 6 weeks of treatment and 2-4 weeks of follow-up) were pooled. For the post-hoc analysis, patients with early stage of schizophrenia (defined as having a disease duration of less than 5 years) were extracted from the whole safety population, and approved doses of cariprazine (1.5-6.0 mg/day) were combined. Treatment-emergent adverse events (TEAEs) and discontinuation rates were analysed versus placebo.

**Results:**

Overall, 169 placebo- (PBO) and 322 cariprazine-treated (CAR) patients were identified as having schizophrenia for less than 5 years. 67.7% cariprazine- and 56.2% placebo-treated patients reported at least one TEAE; most frequently insomnia (10.9 % CAR; 12.4% PBO), akathisia (9.6% CAR; 2.4% PBO) and extrapyramidal symptoms (9.3% CAR; 1.8% PBO). Discontinuation due to adverse events was reported in only 8.4% of cariprazine- and 14.8% of placebo-treated patients. Relapse occurred in 3.1% of cariprazine- and 5.3% of placebo-treated patients.

**Conclusions:**

Cariprazine was generally well-tolerated in the early stage of schizophrenia; given the limitations of this analysis, additional research is warranted.

**Conflict of interest:**

Studies were funded by Gedeon Richter Plc. and Allergan Plc (prior to its acquisition by AbbVie). Dombi, Acsai, Dr. Barabássy, Dr. Sebe, Dr. Laszlovszky, Dr Vass, Dr. Szatmári and Dr. Németh are employees of Gedeon Richter Plc., Dr. Earley and Dr. Patel a

